# Effects of Tropical Climates on the European Constitution

**Published:** 1865-02

**Authors:** 


### EFFECTS OF TROPICAL CLIMATES ON THE EUROPEAN CONSTITUTION.

Mr. Wm. Martin, late Surgeon Bengal Army, in an interesting paper on this subject (*The Medical Mirror*, Oct. 1864,) states:

It is a well established fact, that of those Europeans who make India their residence, a considerable proportion droop and die, or are forced to seek their native air, and with regard to those who become acclimatized, their progeny has, as far as I am aware, in no instance survived to the third generation, *i. e.*, no three successive generations of pure European race have been known to survive. The same, no doubt, would be the case with regard to the natives of tropical climates, who might come to reside in Europe.

"One of the first changes," he remarks, "caused by the removal of a European to a tropical climate, is that of the function of the skin; the perspiration being in most cases greatly increased, sometimes to an inordinate degree. If it be only moderately increased, as is the case of Europeans arriving in India during the winter, when the average temperature may equal that of one of our cool summers, and with those who have become acclimatized, on the approach of each hot season, it affords the greatest relief to the system. An increased amount of perspiration, compared with what obtains in cold regions, for residents in warm climates, must be considered the normal condition. The secretion of the liver is also, in a large majority of cases, increased in the early period of residence, and this is to be considered always as a morbid process to be carefully watched, and, if possible, guarded against; and where it occurs, it must be reduced within due bounds, or it will become a fruitful source of disease, at first functional, eventually, in all probability, organic. It is with respect to this function of the liver that so much caution is required by visitors to warm climates, for on its perversion depends in great measure the amount of derangement of health which occurs among them. On the other hand, the action of the lungs becomes lessened, chiefly if not entirely, in consequence of the increased action of the skin. The great effect this must have, we perceive, when we reflect, that although from the rarefaction of the atmosphere in a hot climate, the lungs must become expanded to a certain extent, yet that this rarefaction is occasioned solely by the increased temperature, and not by diminished atmospheric pressure, as we find to be the case in elevated regions. Consequently the amount of oxygen to be taken in is rather diminished than otherwise, and all the parts concerned in the process of respiration are not called into more vigorous action as they would be in a hilly country, but the reverse; the result of this and of the increased amount of perspiration, is that the work of the lungs is lessened, and this to a considerable extent; so much so, that a most material relief is afforded to the entire system, and if the new arrival be very cautious as to his habits, and particularly the diet, and only so much food of the proper kind be taken as will be digested and assimilated with ease, and the excretions through the lungs, skin, liver and other organs, only task the power of those organs moderately, he may perhaps have nearly as good a chance of preserving health as if he continued to reside in Europe. Should he be naturally inclined to pulmonary disease, the amount of relief to the system afforded in India by the diminished pulmonary action is so great, that he often will enjoy better general health than he did in his native clime, and even will have his life preserved by his change of residence.

The influence of the increased heat of tropical countries upon the skin, in augmenting the amount of perspiration, is so well known, that it does not require expatiating upon; but we may remark that this increase may exist, and often through bad management to a prejudicial extent. It is possible to exceed in the amount of fluid drank; the perspiration, after being inordinately increased, may be suddenly checked; and this counteraction may be in its ultimate effects as dangerous as another condition of the skin, which leads to consequences more directly fatal, in which the perspiration, at a time of excessively sultry heat, becomes suppressed, as is seen to be the case previously to attacks of sunstroke, or insolation; more properly called heat asphyxia. In persons of intemperate habits, an inordinate perspiration is often produced by the very indulgence in intoxicating substances. The system is then left in such a condition that it cannot resist malarious or other noxious agencies; some evil influence will enter the body through the open pores of the cutaneous surface; and the effects of this will be much aggravated by the cooling of the skin, which takes place subsequently, and the rapid contraction of its surface, which renders it incapable of performing its function effectively. In this way seem to arise a large proportion of the deadly diseases so rife in tropical climates; those especially which arise from malaria, also non-malarious dysentery, continued and remittent fevers, cholera, etc.

The liver is, next to the skin, the organ most altered in its action by transference of residence from a cold to a hot climate. Its action is almost always increased to a certain extent, but if great care be taken by paying due attention to regimen, etc., this will pass off, in most cases, in a short time, if the new arrival commences his residence in the hot season, and the skin, with the action of which that of the liver is vicarious, acts freely for a continuance. If he begins his residence in the cold season, he may escape any over-action of the liver altogether; or if it occurs, it will be in less degree, and will be more tractable than in the other case. This increased action is of the nature of functional derangement, and is no doubt attributable to hyperæmia of the organ. This causes at first increased secretion simply, with sympathetic functional derangement of the stomach, and probably of the skin, lungs, etc. If this be speedily checked, and everything is favorable as regards season, and non-malarious condition of the atmosphere, etc., things will return to their original state; otherwise, structural degeneration may occur; but more often than that, there remains a functional derangement of the liver, involving changes of other functions; particularly those with which the liver sympathises; alteration of the constitution of the blood, etc. The derangement is often of such a serious nature, that a proper acclimatization in India is rendered impossible, and change of climate of some kind becomes necessary. In milder cases, the over-action of the liver is succeeded by a corresponding torpor; and this again, while the constitution retains its vigor, by a fit of over excitement; these opposite conditions alternating for some time. Consequently, there is always an irregular and vitiated state of the biliary secretion, with its necessary concomitants, impairment of the nutritive and nervous functions of the body generally. This state of hyperæmia of the liver, although produced in the first instance by increased temperature, is kept up very often by local influences, such as produce malaria. In fact, it exists to a greater extent in comparatively cool weather, as in the rainy and cold seasons in India, than in the hottest. In few cases, however, would the exciting cause act, but for the predisposition caused by the increased temperature. Again, in addition to heat, it seems that there must be some influence which arrests the action of the skin, for it has been remarked that in seasons in which the heat has been great, but without moisture, and consequently in which there has been no impediment to a very free action of the skin, there has been an unusual freedom from congested livers. There is no doubt, however, that long-continued heat, even if dry, will of itself, under certain circumstances, produce a state of hyperæmia.

Acute hyperæmia, or inflammation, often, according to the nature of the exciting causes of disease applied, leads to structural changes, abscess, fatty and other degenerations, etc.; with these may be conjoined the effects of fevers, dysentery, dangerous affections of the kidneys, spleen, etc. Sometimes, there is a protracted condition of chronic hyperæmia, which is too often known only by its effects. The patient experiences nothing perhaps but a general feeling of discomfort, and a state of torpor of the mind and of the functions of the nervous system, and of the principal organs, while organic changes are taking place, which will often be found to be irremediable. Frequently the disease commences in a state of sub-acute hyperæmia, in which there is pain, but not of a severe character, little disturbance of the stomach, only torpor of the chylopoietic functions, with some degree of pyrexia; and this state may merge, according to the nature of any reapplied exciting cause, such as errors in diet, the influence of heat or cold, or wet, or any combinations of these on the patient's peculiar constitution, whether irritable or torpid, into an acute or chronic state of inflammation or hyperæmia. The final results are increase of volume of the liver, sometimes to an enormous extent, or hepatic abscess or exhausting diseases of the bowels; the only chance for saving life being an early change of air, the removal of a European to his native, or at any rate, a milder climate, being, with some exceptions, the most likely means to lead to a restoration of health.

